# Detection of breast cancer using machine learning on time-series diffuse optical transillumination data

**DOI:** 10.1117/1.JBO.29.11.115001

**Published:** 2024-11-11

**Authors:** Nils Harnischmacher, Erik Rodner, Christoph H. Schmitz

**Affiliations:** a HTW - University of Applied Sciences Berlin, Faculty II, KI-Werkstatt, Berlin, Germany; b HTW - University of Applied Sciences Berlin, Faculty I - Health Electronics, Biomedical Electronics and Applied Research (BEAR) Labs, Berlin, Germany

**Keywords:** optical mammography, diffuse optical tomography, machine learning, breast cancer, mammography

## Abstract

**Significance:**

Optical mammography as a promising tool for cancer diagnosis has largely fallen behind expectations. Modern machine learning (ML) methods offer ways to improve cancer detection in diffuse optical transmission data.

**Aim:**

We aim to quantitatively evaluate the classification of cancer-positive versus cancer-negative patients using ML methods on raw transmission time series data from bilateral breast scans during subjects’ rest.

**Approach:**

We use a support vector machine (SVM) with hyperparameter optimization and cross-validation to systematically explore a range of data preprocessing and feature-generation strategies. We also apply an automated ML (AutoML) framework to validate our findings. We use receiver operating characteristics and the corresponding area under the curve (AUC) to quantify classification performance.

**Results:**

For the sample group available (N=63, 18 cancer patients), we demonstrate an AUC score of up to 93.3% for SVM classification and up to 95.0% for the AutoML classifier.

**Conclusions:**

ML offers a viable strategy for clinically relevant breast cancer diagnosis using diffuse-optical transmission measurements. The diagnostic performance of ML on raw data can outperform traditional statistical biomarkers derived from reconstructed image time series. To achieve clinically relevant performance, our ML approach requires simultaneous bilateral scanning of the breasts with spatially dense channel coverage.

## 1 Introduction

Breast cancer is the most prevalent of all cancer types and a leading cause of death among women.^1^^,^^2^ Early detection of malignant breast lesions is generally accepted as the best route to maximize treatment success and improve the prognosis of the disease.^3^ The clinical standard for breast cancer screening is X-ray mammography, despite some of its disadvantages, especially its use of ionizing radiation and its limited diagnostic success in radiologically dense breasts, the younger population, and early-stage tumors.^4^ A comprehensive breast cancer detection strategy therefore includes other modalities such as palpation/self-exam and other established imaging techniques, including ultrasound and MRI.^5^^,^^6^

Starting in the mid-1990s, diffuse optical imaging has been widely investigated as a potential adjunct or alternative to X-ray mammography screening, expecting that it would lead the path to detecting tumors earlier and in a wider population, using technology that is inherently safe, compact, and low cost. In particular, the prospect of exploiting spectroscopic tissue contrasts is seen as a promising way to reveal metabolic processes and tissue function, thereby providing improved diagnostic value compared with anatomical imaging techniques.^7^^,^^8^

However, diffuse optical mammography has always suffered from severe practical challenges that are rooted in intrinsically weak optical contrasts, the reproducibility of contact-based diffuse optical measurements on highly irregular targets, and the ill-posed, underdetermined image reconstruction problem. Innovative developments have been made over the years to ameliorate these challenges, including the use of short-pulsed^9^ or radiofrequency-modulated light,^10^ the application of increasingly cheap computational power, and the use of extrinsic contrast agents.^11^ Despite these methodological advances and several large-scale clinical studies, diffuse optical breast imaging has never entered a common clinical practice in radiology. A comprehensive review report on optical breast cancer imaging from 2016 concluded that “Currently, sensitivity and specificity of optical mammography are likely too low for its application as a screening tool.”^8^ An earlier meta-study by Leff et al.^12^ found that “The current analysis suggests that O[ptical] M[ammography] is capable of detecting up to 85% of lesions. This value is comparatively low considering that the majority of studies have involved retrospective comparisons with X-ray mammography.”

Lately, research activity both in method development and clinical studies of optical breast imaging as a screening tool seems to have declined, but there remains interest in its multi-modal integration with established imaging methods.^13^^,^^14^ A study by Petrillo et al.^15^ used single-breast transillumination and mild tissue pressure modulation to track perfusion changes as an indicator of breast malignancy. Using optical biomarkers alone, they reached 0.63 area under the curve (AUC) of the receiver operating characteristics (ROC); in combination with mammography and ultrasound, they achieved 0.73 AUC and 0.96 AUC. There is also continued interest in optical monitoring of neoadjuvant chemotherapy^5^^,^^16^^–^^18^ and tumor classification.^17^^,^^19^ A review by Poplack et al.^7^ concluded that there is promise for optical breast imaging expected from “novel engineering techniques and AI.”

One attempt at enhancing the diagnostic performance of diffuse optical mammography is the acquisition of a rapid sequence of images and the use of time series analysis to extract tissue dynamics, thus taking advantage of the physiological information carried by vascular dynamics. Tumors exert strong disturbances on the vascular integrity^20^ and the autoregulatory response of the diseased tissue and its surroundings, which leads to distinct spatio-temporal signatures in the diffuse optical data.^21^ This approach of dynamic optical tomography was pioneered in the early 2000s by developing the required instrumentation,^22^^–^^24^ image reconstruction methods,^25^^,^^26^ and diffuse optical imaging time series analysis tools.^27^^–^^29^

The instrumentation was subsequently expanded to allow simultaneous, bilateral, and parallel scanning of both of the patient’s breasts to achieve a subject-specific self-referencing of the measurement.^30^

Bilateral dynamic diffuse optical imaging of the breast has been demonstrated to discriminate between healthy and tumor-bearing breasts in a patient study.^28^^,^^29^ Because these reports came out soon before and after the above-mentioned review article by Grosenick et al.,^8^ the markedly improved diagnostic performance of bilateral optical breast imaging did not enter into the reviewed statistics.

Spurred by the advances in artificial intelligence and machine learning (ML) in recent years, we have seen a new trend of applying these strategies to optical breast imaging for a wide range of uses, including the improvement of image reconstruction,^31^^,^^32^ to extract optical properties,^33^^,^^34^ to classify diffuse optical images,^35^^–^^38^ and to stage lesions.^39^ Recently, the application of ML methods to dynamic optical breast imaging has been reported for the case of single-breast planar transillumination.^39^

In this paper, we evaluate the merit of analyzing dynamic bilateral tomographic optical breast scans with ML methods and compare the results to diagnostic findings previously obtained with model-based statistical methods. To our knowledge, this is the first time that ML methods have been applied to this imaging modality.

## 2 Methods

### 2.1 Instrumentation

We investigate a time series of diffuse optical data that were obtained on breast cancer patients and healthy controls. The data were acquired with a diffuse optical tomography instrument operating in a continuous-wave mode that performs simultaneous bilateral transillumination of both breasts at a frame rate of ∼1.8  Hz. The imaging setup’s fiber optic tissue interface allows the placement of 32 emitters and 64 detectors on each breast for a wide range of anatomical geometries and sizes. The opto-electronic architecture of the device achieves an exceptionally high dynamic measurement range and allows full tomographic transillumination of both breasts at two wavelengths (760 and 830 nm), with 32×64=2048 channels for each side. The instrumentation has been described in more detail in previous reports.^30^^,^^40^

### 2.2 Patient Study

The clinical study was conducted at the State University of New York Downstate Medical Center, Brooklyn, New York, United States, from November 2008 to June 2011. A total of 63 female volunteers participated in the study, 18 of whom were diagnosed with breast cancer, and the rest of whom showed no or non-cancerous pathologies of the breast. Detailed clinical information about the enrolled subject group, the study design, and the ethical aspects of the study were described in detail in a previous report.^28^ To ensure patient confidentiality, all personal patient information in the data files or accompanying metadata was removed or replaced by randomized codes before datasets were made available to the authors.

### 2.3 Data Collection

For the acquisition of the optical time series, each subject was seated in front of the fiber optic interface, which was then positioned on both breasts. Each imaging session started with a resting period of at least 400 time frames (equivalent to 220 s) duration, which was followed by a series of different provocation protocols to elicit hemodynamic activity in the peripheral vasculature. The full data collection procedure is described by Graber et al.^28^ Similar to the authors of this study, we only considered the resting-phase data. Unlike provocation responses, resting state data are much less affected by external factors such as the patients’ ability to comply with maneuver instructions or the generation of motion artifacts and thus generally offer much more consistent measurements. Datasets for which the initial rest phase exceeded a duration of 400 time frames were truncated to avoid biases due to uneven lengths of the feature vectors.

**Fig. 1:**
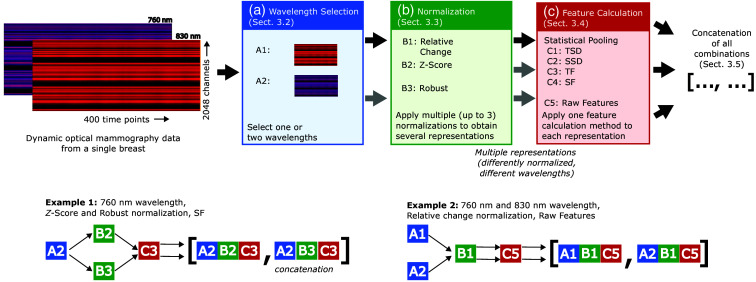
Overview of our pipeline for calculating unilateral data representations: the pipeline consists of three steps (A, B, and C) with different options resulting in various representations that we combine afterward. The examples illustrate how multiple unilateral single-feature representations of the data are combined into concatenated representations.

Hemodynamic activity in the breast is most prominent in the very low frequency range, below 0.1 Hz (see figures 1 and 2 of Graber et al.^28^). These rhythms are associated with the vasomotion, which is hypothesized to be primarily impacted by neoplastic disease and which is expected to be a main contributor to any time-series-based cancer classification. To adequately capture this frequency range, data need to be acquired for at least the duration of the associated periods, which is on the order of tens of seconds to a minute. We therefore opted to use the maximum amount of data available to us, ∼3  min and 40 s, which we judged optimal for capturing the desired low frequency range. We did some preliminary exploratory studies in which we looked at the effect of using shorter time series on the classification result, and we observed a decline in performance for data series length below approximately 100 data points, as one would expect. Because a rigorous, physiology-driven argument can be made for using the maximum amount of data available, and to maximize the chance of hemodynamic-based disease classification, we opted for only working with the full 400-time-point series.

### 2.4 Statistical Cancer Biomarkers

The authors of the original study formulated scalar metrics based on the tempo-spatially varying hemoglobin (Hb) and oxy-hemoglobin (HbO) concentration changes in the reconstructed volumetric image time series, which are shown to yield biomarkers for the detection of breast cancer.^28^

To obtain metrics of hemodynamic spatio-temporal heterogeneity, they computed the arithmetic mean and the standard deviation along the temporal or the spatial dimensions of the image time series and combined pairs of those into scalar metrics, e.g., the spatial mean of the temporal standard deviation, or the spatial standard deviation of the temporal standard deviation. Overall, six metrics can be constructed in this fashion and were evaluated in their work with univariate and multivariate tests to judge their predictive performance for detecting breast cancer. In this paper, we tested the usefulness of applying these metrics to ML by combining them into a single feature vector, referred to as “six features” (SF).

Graber et al. investigated the benefit of acquiring data bilaterally and simultaneously on both subject’s breasts. The rationale for this is that symmetric bilateral breast cancer is rare and that the anatomical symmetry of the breasts offers within-subject referencing of the data, which can help overcome the notorious difficulty of achieving reproducible measurements between subjects and/or scans using optical measures. Their paper evaluates the diagnostic performance of unilateral metrics that classify each breast as cancerous or non-cancerous, as well as bilateral metrics that seek to classify patients as cancer-positive or negative, based on measured differences between their breasts.

## 3 Machine Learning Pipeline for Spatiotemporal Optical Mammography Data

As guidance through this section, Fig. 1 shows an overview of our pipeline for extracting data features and generating representations that can be used in the ML classification. In the following, we describe the steps involved in this process in detail.

### 3.1 Preliminaries

Each single data point within the time series represents the voltage produced by an optical detection circuit at time t∈[1,400] (time steps within the resting phase, see Sec. 2.3) for the measurement channel r∈[1,2048] (64×32 detector-emitter pairs, see Sec. 2.1), at wavelength λ∈{760  nm,830  nm}, and the breast side b∈{left,right}. Consequently, we denote our 4D dataset as follows: ${\tilde{x}}_{b}^{\lambda }(r,t)$. The goal of our paper is to show the capabilities of ML methods for classifying high-density, time-varying diffuse optical transmission data. We therefore avoided any analysis strategies that are guided by physiological insights and only applied minimal pre-processing of the data. Instead, we defined a variety of ways to transform the high-dimensional information of the datasets into vector representations, as further explained in Sec. 3.4. These representations of the data or even combinations of them are then subjected to classification with ML.

### 3.2 Wavelength Selection

The first step involves selecting the wavelengths (see process step A in Fig. 1). This allows us to experimentally evaluate which of the wavelengths leads to better tumor classification, i.e., which raw data contain the most discriminative information.

To test the benefit of dual-wavelength measurements, we also fused the single-wavelength data into a single representation (see Sec. 3.5 for details about our fusing strategy).

### 3.3 Normalization

The second step in our processing pipeline is the normalization of the data to account for the fact that the imaging method does not provide a measure of absolute (i.e., calibrated) quantities of transmitted light intensity, and therefore, only relative variations in the readings can be analyzed. Normalization is a common step when analyzing diffuse optical data to be able to relate data across channels, measuring sites, and patients.

We evaluate four data normalization methods, which were chosen from a range of approaches commonly encountered in ML and in diffuse optical tissue imaging. Each one, or any combination of these, can be evaluated by incorporating them into vector representations of the data.

1. Relative change, i.e., normalization of each data point to the global mean of the dataset
  $$x_{b}^{\lambda }(r,t)=\frac{{\overset{˜}{x}}_{b}^{\lambda }(r,t)}{\mu ({\overset{˜}{X}}_{b}^{\lambda })},$$ (1)
2. Z-score, i.e., normalizing the standard deviation
  $$x_{b}^{\lambda }(r,t)=\frac{{\overset{˜}{x}}_{b}^{\lambda }(r,t)-\mu ({\overset{˜}{X}}_{b}^{\lambda })}{\sigma ({\overset{˜}{X}}_{b}^{\lambda })},$$ (2)
3. zero centering by subtracting the mean
  $$x_{b}^{\lambda }(r,t)={\overset{˜}{x}}_{b}^{\lambda }(r,t)-\mu ({\overset{˜}{X}}_{b}^{\lambda }),$$ (3)
4. robust normalization, similar to z-scoring but less sensitive to outliers
  $$x_{b}^{\lambda }(r,t)=\frac{{\overset{˜}{x}}_{b}^{\lambda }(r,t)-\text{median}({\overset{˜}{X}}_{b}^{\lambda })}{p^{75}({\overset{˜}{X}}_{b}^{\lambda })-p^{25}({\overset{˜}{X}}_{b}^{\lambda })}.$$ (4)

In the above expressions, $\mu ({\overset{˜}{X}}_{b}^{\lambda })$ and $\sigma ({\overset{˜}{X}}_{b}^{\lambda })$ refer to the global mean and standard deviation of the dataset, respectively. Here, $p^{75}({\overset{˜}{X}}_{b}^{\lambda })$ is the third, and $p^{25}({\overset{˜}{X}}_{b}^{\lambda })$ is the first quantile of the data.

### 3.4 Feature Calculation: Raw-Feature Vectorization or Statistical Pooling

To subject the normalized two-dimensional data to ML, we define various ways of extracting information and defining single vector representations.

A straightforward option is to flatten the normalized time series data into a single vector. The length of this vector for a single wavelength per breast is 32 emitters ×64 detectors ×400 time points = 819.200 entries. Because no further statistical pooling takes place, we refer to this as “raw features” (RF).

Although this method preserves the full information content, it leads to large feature vectors, which can result in severe over-fitting, especially for our small medical datasets. Alternatively, one can seek to reduce the dimensionality of the data representation by defining metrics that aggregate information, for example, according to specific expectations that may be informed by the problem at hand. In our case, one expects a tumor to affect the autonomous hemodynamics of the breast in ways that cause more irregular and inhomogeneous behavior both in the spatial and temporal domains. Therefore, the calculation of simple statistical metrics, such as the mean or standard deviation, can reveal such behavior, as had been shown previously by Graber et al.^28^ Following their work, we considered four statistical representations of the data: The temporal standard deviation (TSD) of a dataset, its spatial standard deviation (SSD), and the concatenation of the two (TF). TSD and SSD are vectors of dimensions 32×64=2048 and 400, respectively, and TF has the length 2048+400=2448. We furthermore included the six scalar spatio-temporal metrics that can be derived from all combinations of first calculating the mean or standard deviation of first one domain (space or time) and then the other, as had been introduced by Graber et al.^28^ We combine the six resulting scalars into one feature vector, referred to as SF (see Sec. 2.4).

### 3.5 Feature Concatenation for High-Dimensional Unilateral Representations

To optimize the classification results, the described features can be concatenated into longer feature vectors, each of which forms a specific representation of the data. This is often referred to as early fusion, whereas late fusion refers to fusing the outputs of different models working on individual representations. In contrast to late fusion, a single model based on early fusion can exploit the correlations between feature representations.

To derive a single feature representation, we subject our data to the following sequence of data processing steps (Fig. 1):

1. selection of the considered wavelengths: 760, 830 nm [Sec. 3.2, Fig. 1 A]
2. normalization of the data with one of the methods described in Sec. 3.3: relative change, Z-score, zero-centering, or robust [Fig. 1 B]
3. statistical pooling or vectorization [Sec. 3.4, Fig. 1 C].

This results in different feature vectors depending on the design choices selected in each step. The process is done for each breast, so we denote the ℓ’th unilateral feature as $X_{b}^{\ell }$. These can then be further concatenated into multi-feature data representations. The representation resulting from concatenating L unilateral features is given by the unilateral representation $X_{b}=[X_{b}^{1},\ldots ,X_{b}^{L}]$.

Using this approach, we can build 210 different unilateral representations. We use a cross-validation technique further described in Sec. 4.1 to benchmark which data representation yields the best classification and hence diagnostic performance.

### 3.6 Feature Distances for Bilateral Representations

To evaluate the benefit of bilateral breast imaging, we developed a feature representation that is sensitive to differences in data obtained from both breasts. This bilateral feature is readily subjected to ML, thus effectively performing a normalization or comparison between breasts without the need for running ML on datasets from different breast sides separately.

Our bilateral feature representation is obtained by calculating the Euclidean distance between the same two unilateral features ℓ for both breast sides

$$d_{\ell }=\|X_{\text{right}}^{\ell }-X_{\text{left}}^{\ell }\|,$$ (5)

which can be further concatenated to generate bilateral multi-feature representations

$$\overset{¯}{X}={\left[d_{1},\ldots ,d_{L}\right]}^{T}.$$ (6)

We chose this bilateral feature representation as a way of reducing the dimensionality of the dataset while preserving any feature that might be indicative of an imbalance of tissue characteristics in the two breasts.

### 3.7 Machine Learning Model Selection

Because of the high dimensionality of the raw data and the feature vectors, we chose a linear support vector machine (SVM) as the primary model for our tests. To evaluate whether more complex models (including non-linear models and ensembles) could further improve the performance, we also tested the AutoML framework AutoGluon.^41^ AutoML techniques perform model selection for a large set of candidates in an efficient manner and with a user-provided time budget. In our case, this allows for systematic and efficient testing of multiple models and model architectures.

### 3.8 Simulating Sparse Spatial Sampling

The dataset investigated in the present study was generated with an array of 32 optical sources and 64 detectors that were closely spaced around the entirety of the breast, thus affording full angle-of-view transmission measurements at a high density. Although this setup is highly beneficial for diffuse-optical tomographic image reconstruction, it puts high demands on the imaging hardware because of the required large number of sources and detectors and the need for a very high dynamic measurement range. We sought to evaluate whether ML-based data analysis benefits from such high-density imaging arrays or whether sparser imaging setups might also prove adequate for classifying the presence of breast malignancies. To test the effect of channel density on the classification results, we applied our ML pipeline to subsets of the original data, in which source and/or data channels were removed to simulate the use of a reduced hardware setup. The full dataset for one breast consists of 32 sources by 64 detector channels, from which we derived subsets of varying channel counts, namely, 32×32, 16×16, 8×8, and 4×4 sources by detectors, respectively. Whereas mathematically there exist a large number of subsets, for each given number of sources and detectors there are only a limited number of arrangements that are useful, i.e. that allow transillumination of a large aspect of the breast. To achieve this, the probes should be arranged in a homogeneous spatial distribution, avoiding local clustering through close spacing of probes in some areas while leaving others void of probes. We therefore manually created sparse optode arrangements with the available positions that would mimic the way in which one would create an imaging field for a given number of fewer sources and detectors *de novo*.

Because the sparse grids are subsets of the full 32×64 positions, the simulated sparse geometries are confined to positions available on the 32×64 grid. One particular property of the imaging setup is that all 32 source positions are also 32 co-located detector positions. Additional 32 detector positions are interspersed between these. We therefore have the opportunity to create sparse grids in which there are co-located source-detector pairs, and grids where sources and detectors are spatially disjunct. The sparse grids were manually curated to obtain as even a tissue coverage as possible within the constraints posed by the full grid. We identified two geometries with 32 sources and 32 detectors, three geometries with 16 sources and 16 detectors, and four geometries with 8 or fewer sources and detectors. The spatial arrangement of the full grid and the selected sparse grids are shown in the Supplementary Material.

## 4 Machine Learning Evaluation Setup

In the following, we provide details of our evaluation setup, including our data splitting strategy as well as the performance measures used.

### 4.1 Model Selection and Testing Strategy

**Fig. 2:**
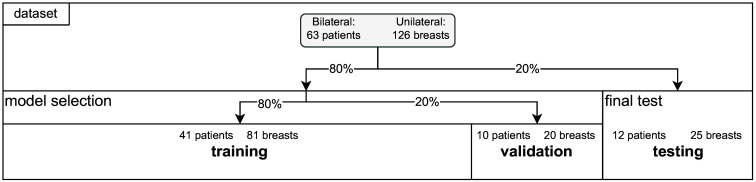
Illustration of our data split into model selection and testing sets.

Model selection involves SVM hyperparameter optimization as well as feature representation selection. For the selection procedure itself, we use standard cross-validation with 20 overlapping stratified shuffle splits and use 20% of the data for validation. Figure 2 provides an overview of our data splitting strategy. Our dataset consisted of bilateral breast measurements from 63 patients. For our unilateral analyses, measurements from each breast are treated as independent records, yielding 126 measurements. Following the standard protocol in ML, we split our data into two disjoint sets: “model selection” data (101 breast measurements, 80%) and “final test” data (25 breast measurements, 20%). This follows the typical trade-off of training and test sizes also used in common fivefold cross-validation.^42^ The model selection set was further split several times into “training” (80%) and “validation” (20%) sets of 81 and 20 breast recordings, respectively. Please note that due to our splits being based on percentages of the overall sample sizes, the left and right breasts of a single patient were assigned to different subsets.

For bilateral analysis, data from both breasts are analyzed for each of the 63 patients. Following the percentage-based split strategy of the unilateral case, we obtain sample sizes of 41, 10, and 12 patients for training, validation, and testing, respectively.

Please note that while cross-validation is used for model selection, we use a single predefined test set for evaluation. This was done to significantly reduce the computation effort of our experiments, which would otherwise scale quadratically in the number of folds.

### 4.2 Performance Measures

Breast cancer detection from optical mammography data is a binary classification task. Therefore, we use ROC curves for evaluating the performance of our approach. In contrast to classical accuracy or F1-score measures, ROC curves are independent of the decision threshold that would have to be defined for a specific application of mammography. For the purpose of comparing different classification results, we calculate the area under the ROC curve (AUC). We do not present sensitivity/specificity values because our data split leaves us with comparatively few data points and strongly discretized ROC curves. The selection of a diagnostic threshold for determining sensitivity and specificity would inevitably underestimate one and overestimate the other. Furthermore, AUC has a clear probabilistic interpretation—it states the probability that the scores of two random examples are ordered according to their label, e.g., a negative example has a lower prediction score than a positive example.^43^

## 5 Results

In the following, we present and discuss our results both for the unilateral and bilateral cases. Furthermore, we investigate our design choices and the performance of a reduced hardware setup by simulating it.

### 5.1 Comparison of Bilateral and Unilateral Classification

Table 1 summarizes the classification results obtained with various feature representations of the optical transmission data. For brevity, we only report the most successful strategies for unilateral and bilateral imaging, in both cases for single and concatenated representations.

**Table 1:** Comparison of the best-performing single- and multi-feature data representations for unilateral and bilateral classifications.

Representation	AUC (%)	Data processing steps
Single representation, unilateral	55.1	A: 830 nm; B: robust normalization; C: TSD
Concatenated representation, unilateral	79.7	A: 760 nm; B: relative change + robust normalization; C: SF
Single representation, bilateral	80.0	A: 830 nm; B: relative change C: SF
Concatenated representation, bilateral	93.3	A: 760, 830 nm; B: relative change + zero-centering; C: RF

We first tested our so-called “single representations” $X_{b}^{\ell }$, i.e., processing permutations in which we would apply only any one of steps 1 to 3 of our processing pipeline (Sec. 3.5). For the unilateral analysis, this approach was marginally better than chance level, with an AUC value of 55.1%. This result was achieved for a wavelength of 830 nm, using robust normalization and TSD as the feature calculation. When combining individual features into concatenated representations $X_{b}$, the classification results improved, with the most successful data representation reaching 79.7% AUC. This was achieved using 760 nm data, concatenating the results from relative change normalization and robust normalization, and using SF feature calculation.

For bilateral analysis, the best single representation $d_{\ell }$ yielded 80.0% AUC when using 830 nm data, normalizing these with relative change and using SF feature extraction. The best of the bilateral concatenated representations $\overset{¯}{X}$ achieved 93.3% AUC when using both wavelengths, applying “relative change” and “zero-centering” normalization, and using RF vectorization.

### 5.2 Comparison of Different Wavelength Selections

To gain an understanding as to which imaging parameters may contribute to classification success, we studied the influence of the selected wavelength combination, i.e., using only 760 nm, only 830 nm, or both. The results for the best-concatenated representation in each case are summarized in Table 2. For unilateral and bilateral classification, 760 nm outperformed 830 nm when considering single-wavelength representations. Using both wavelengths did not improve the classification results; for the unilateral case, it actually reduced the AUC value compared with the single-wavelength representation; in the case of bilateral classification, the dual-wavelength result was on par with the best single-wavelength classification.

**Table 2:** Comparison of the best performing unilateral and bilateral representations for different wavelength combinations.

	Wavelength (nm)	AUC (%)
Unilateral	760	79.7
	830	66.6
	760 & 830	78.2
Bilateral	760	93.3
	830	90.0
	760 & 830	93.3

### 5.3 Comparison of SVM Classification with Statistical Biomarkers

**Fig. 3:**
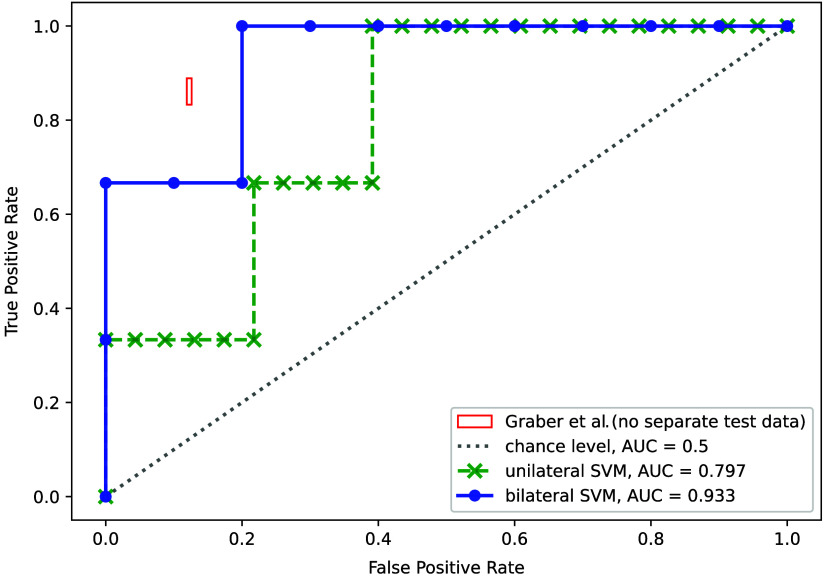
ROC curves of our best-performing unilateral and bilateral classifications in comparison to the performance of the best multivariate statistical biomarker by Graber et al.^28^ Their results are shown as a box to represent the ranges of sensitivity and specificity values that they obtained for all subjects.

Figure 3 shows the ROC curves for the best unilateral and bilateral classification results from Table 1. Also shown for comparison is the best result of the hypothesis-driven statistical analysis by Graber et al. on the same dataset (first row of table V in Ref. 28). For this, they did not report any data splitting for separate training and testing, so we assume that it is a best-case outcome. Because they published no ROC curve, we indicate their reported ranges of 83.3% to 88.9% and 87.4% to 88.1% for sensitivity and specificity, respectively. Their reported range of AUC values for these results is 86.3% to 86.9%.

### 5.4 Comparison of Linear and Non-linear Classification

**Fig. 4:**
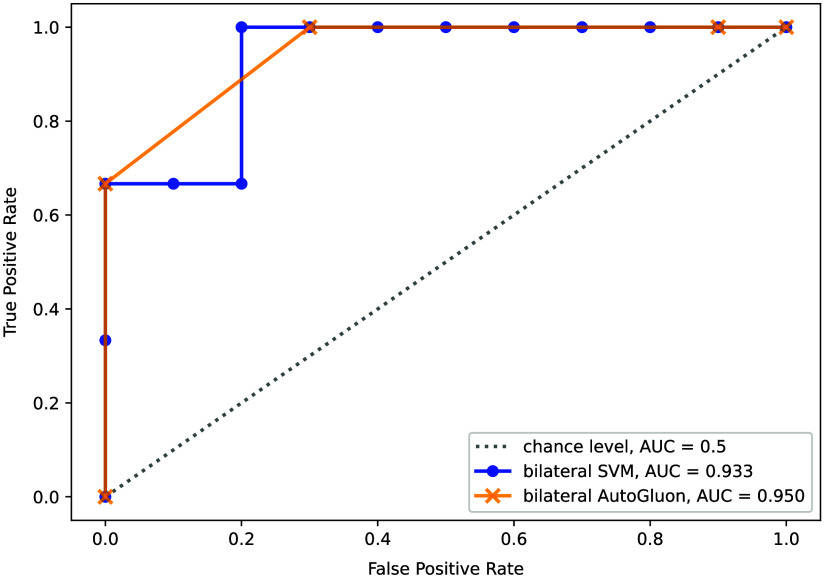
Comparison of the ROC curves achieved by our SVM model and an AutoGluon model, using our best bilateral, multi-feature data representation.

For our study, we used a linear SVM classifier because of its conceptual simplicity, robustness, and computational efficiency. In addition, we wanted to use our most successful data representation with more complex, non-linear classifiers to evaluate whether our SVM results could possibly be improved. The model ensemble created by AutoGLuon reached an AUC value of 95.0%. The corresponding ROC curve is shown in Fig. 4 in comparison to the SVM result.

### 5.5 Influence of Channel Density on Classification Performance

**Fig. 5:**
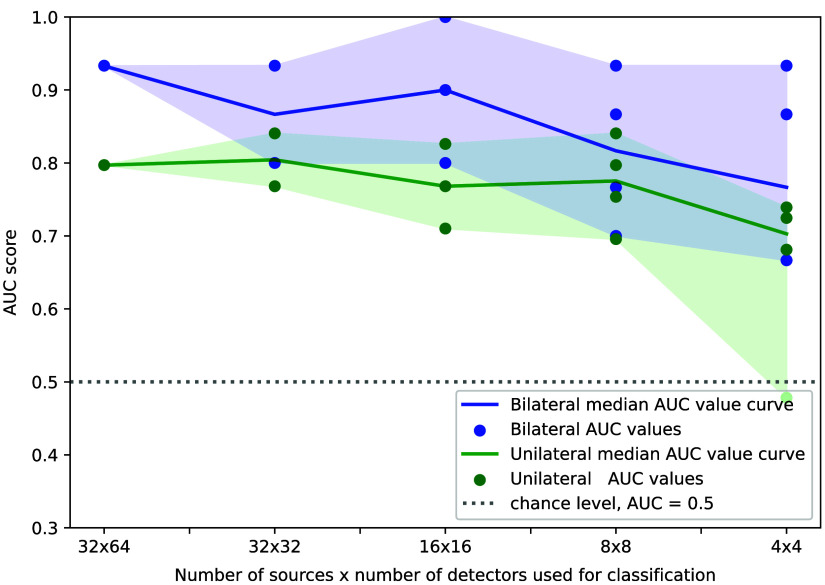
Classification results for varying numbers of source-detector channels.

Figure 5 shows the effect of sparsifying the measurement data on SVM classification results for our most successful unilateral and bilateral data representations. When sparsifying the dataset, there exist multiple distinct sets of data channels with (near-) homogeneous spatial coverage of the breast. For a reduced dataset of 32×32 channels, we identified two; for 16×16 channels, we identified three suitable measurement geometries; and for the other reduced datasets, we found four useful distinct arrangements in each case. Plotted are the AUC values for each of the tested arrangements, and the line plots indicate the median AUC value for each arrangement. The shaded areas enclose the regions between the minimum and maximum values for each case.

## 6 Discussion

We have, to our knowledge, for the first time applied ML methods to time series of multi-channel, diffuse optical transillumination data obtained simultaneously from both breasts of female subjects with and without breast cancer.

Our work capitalized on the availability of a particularly information-rich data set that contains long-duration volumetric hemodynamic signatures captured synchronously and with dense spatial sampling from both breasts of patients undergoing clinical breast cancer diagnosis. The instrument used for obtaining the data was uniquely designed to provide the required technical capabilities. The data set from the original study comprised experiments from 63 patients, which can be considered on the low end of the amount of data desired to train and test ML methods. No additional or more recent data with the identical instrumentation setup and experimental protocol has become available since, and we adopted widely accepted analysis strategies for dealing with limited data sets. Because one of the main objectives of our study was the comparison of modern ML tools with hypothesis-based statistical models, we were confined to working with the existing data. Having access to a less-than-ideal amount of data is a typical challenge in ML, and methods have been devised to minimize fitting errors and maximize confidence in the classification result. To deal with the limited amount of data sets available to us, we followed the standard procedure of strictly separating model selection from final model evaluation (cf. Sec. 4.1). We furthermore made sure to adhere to best practices by complying with standards as best as possible issued by the TRIPOD statement initiative.^44^

To deal with the large dimensionality of the data and the associated risk of overfitting, we defined a range of data features and representations that were tested both with a linear classifier (SVM) and an automated ML framework (AutoGluon) that applies a wider range of models, including nonlinear approaches. We decided on an SVM model as our primary ML method for investigating the influence of various data features on the classification success because these classifiers are well understood, offer robust behavior, and lend themselves to easy interpretation of the results. The AutoGluon model, which is more of a black-box approach, was used to independently validate the main findings obtained with SVM.

Our SVM model was able to classify the conditions “cancer-positive” versus “cancer-negative” with a performance of 93.3% AUC of the ROC curve. This result supersedes previously published outcomes of 86.9% AUC, obtained with hypothesis-driven statistical models on the identical dataset, by 6.4%.^28^ The AutoGluon model confirmed this tendency and performed even slightly better (95.0% AUC), exceeding the best result of Graber et al. by 8.1%.

We found that classification outcome is strongly influenced by variations in preprocessing and the generation of data representations. Investigating these influences allows us to determine which data features have the best discriminatory power and, from this, to infer which strategies for designing instrumentation, collecting data, and defining feature representation may hold the most promise when considering future works.

Even though, to the extent possible, we rationalize the success of certain data properties (e.g., number of wavelengths, unilateral versus bilateral) after the fact; one should keep in mind that the black-box nature of the ML approach does not allow any conclusion about causal relation between the data and the classification performance and therefore allows no general prediction about the success of a particular feature.

### 6.1 Influence of Single- Versus Multi-Feature Representations

We define a single-feature data representation as one specific combination of several processing steps consisting of wavelength selection, data normalization, and feature calculation (see Fig. 1). We tested these single-feature representations individually, as well as multi-feature data representations that we obtained by concatenating the single-feature vectors.

Table 1 compares the performance of single- and multi-feature representations for unilateral and bilateral breast cancer detection. We observed that, generally, multi-feature representations show clear improvement over single-feature results. For the unilateral metrics, single feature representations reached 55.1% AUC, barely exceeding the chance level. When combining multiple unilateral features into a concatenated representation, the performance markedly improved to 79.7% AUC. For bilateral analysis, the single feature representation reached 80.0% AUC, whereas multi-feature representations reached 93.3% AUC, the best overall performance of our SVM classifier.

### 6.2 Unilateral Versus Bilateral Classifiers

The single biggest impact on classification performance was the implementation of features that were designed to be sensitive to differences in the spatio-temporal signatures of the datasets from two breasts of a subject (i.e., “bilateral” feature representations).

The rationale of simultaneous bilateral sensing of tissue dynamics in the breasts is the realization that the unilateral presence of a tumor causes an imbalance in the hemodynamic behavior that can be detected with a higher degree of reliability than the deviation from some baseline behavior of a single breast.^28^^–^^30^ Unilateral data representations treat each breast as an independent measurement, and the classification success relies on the presence of characteristic spatio-temporal patterns that would be caused by a tumor. Given the small sample size and the variety of tumor sizes, types, stages, and locations, it comes as no surprise that we found unilateral classification consistently inferior to bilateral metrics.

Figure 3 summarizes the comparison of the two ROC curves for our best uni- and bilateral representations. The relatively small number of datasets and the required splitting of data into model selection and testing sets lead to strongly discretized shapes, which precludes the identification of sensible sensitivity/specificity pairs. Throughout this paper, we therefore only use AUC values to compare our classification results among each other or with findings from other studies. To compare our findings with Graber et al.,^28^ we show their best classification result, which is a bilateral, multivariate metric. They do not present ROC curves but report ranges of AUC, sensitivity, and specificity values for all subjects. We therefore indicate their result with a box that covers the ranges of sensitivity (83.3% to 88.9%) and specificity (87.4% to 88.1%) that they reported for all subjects. It is important to note that the result of Ref. 28 was obtained by an overall data analysis without proper splitting into training, validation, and test data to avoid overfitting and therefore has to be assumed to be a best-case estimation.

Despite the coarse shape of our ROC curves, we observe that our unilateral results do not come close to the results obtained with bilateral statistical data evaluation by Graber et al. Our bilateral SVM results, however, reach far up into the upper left corner of the characteristic, and it is reasonable to assume that this could well have fallen within the vicinity of Graber’s result, had we obtained more test data and a smoother ROC curve. This further confirms the validity of both analyses and the overall validity of bilateral dynamic optical breast cancer detection.

To further validate our findings, we generated the ROC curve for the bilateral AutoGluon model, which we show in comparison to the results of our bilateral SVM model in Fig. 4. Both curves are of largely identical shape, apart from their behavior in the upper left corner of the graph. Because of the coarse discretization of both curves, we can make no quantitatively sound comparisons of the shapes of both curves but rather conclude that both characteristics show largely consistent behavior.

Our findings support the conclusion of the original study by Graber et al.^28^ that bilateral metrics are far superior for detecting the presence of breast cancer because confounding physiological and experimental factors unrelated to the presence of cancer tend to cancel one another out. We conclude that future dynamic optical mammography studies should be carried out bilaterally and synchronously for both breasts to maximize the chance of tumor detection.

### 6.3 Wavelength Selection

Our data representations are constructed in such a way that the preprocessing and feature calculations are applied to the transmission data for a single wavelength (760 or 830 nm), which are then either evaluated separately or concatenated into a dual-wavelength representation of the data. This approach allows us to evaluate which of the two wavelengths, or their combination, offers the greatest discriminatory power for a given set of preprocessing and feature definition steps.

Our results are summarized in Table 2. With unilateral classification, the best result is achieved for a single wavelength of 760 nm (79.7% AUC), whereas the use of 830 nm yielded a markedly lower performance of 66.6% AUC. Using both wavelengths in a unilateral classifier reached 78.2% AUC, a slightly lower performance than 760 nm alone. Although it may seem surprising that added information may lead to lower classification success, this can be explained by the fact that we are now combining a good classifier (760 nm) with a poorer one (830 nm), so the latter can act to “dilute” the discriminatory information of the former by introducing what amounts to informational noise in the data. This indicates that there is, in fact, less discriminatory power in the longer wavelength, even though the raw data from the two wavelengths of diffuse optical tissue measurements usually show a high correlation. Whether such informational loss in the higher wavelength is due to methodological or instrumental origin, or whether this has a physiological origin, is unknown to us. Due to the black-box nature of ML analysis, the classification performance of a feature allows no causal inference about the underlying properties of the data. Why a certain wavelength combination would perform better than another one therefore cannot be determined, and it is difficult to draw general conclusions or make predictions. The inconsistent results obtained with different wavelengths on the raw intensity data may also suggest that further data preprocessing, especially filtering and the calculation of Hb states, may serve to improve the information-to-noise of the data before subjecting these to ML methods, potentially further improving classification results.

For the bilateral classifiers, the performance is much better than for the unilateral ones and also more consistent for the different wavelength combinations, which all score in the lower 90% region. Again, 760 nm outperforms 830 nm, but only barely (93.3% versus 90.0%), and dual-wavelength classification scores identical to 760 nm. These results suggest that data for both wavelengths carry about the same information content for a bilateral classifier.

The greater discrepancy between the unilateral results and their overall lower performance compared with bilateral ones is further evidence that unilateral optical breast imaging is challenged by inter-subject and inter-measurement variables that confound the detection of signal components characteristic of the presence of a tumor. One could hypothesize that it is the hypoxic state of tumors that causes the greater sensitivity of the shorter wavelength due to the much greater absorption of reduced Hb in this spectral region, as well as the overall greater attenuation. However, there is no way to substantiate such claims within the scope of this work. Even if we could, this could mean that unilateral metrics are probably more sensitive to certain classes of tumors (i.e., higher stages) than others. Although this in itself may be a feature to be exploited for diagnostic purposes, for the basic task of screening for the presence of cancer, it is paramount to catch all the stages, particularly early ones.

Bilateral classifiers, in contrast, appear to be working about equally well for all wavelength combinations. This further supports the hypothesis that *any* imbalance in the data serves to predict the presence of a tumor, independent of any specific physiological premise, such as tumor stage or size.

One conclusion from these findings is that it may be sufficient or even beneficial to use a single wavelength for ML-based optical breast cancer detection. In unilateral cases, this may serve to maximize the classification success for specific types of tumors. For bilateral classification, 760 nm alone performs just as well as two wavelengths, which may motivate the design of single-wavelength instruments in forthcoming development efforts, thereby reducing system complexity and costs.

It should be noted that our study analyzed readings of transmitted intensity rather than reconstructed time series images of Hb concentration. Our approach affords a more direct evaluation of the measured data and avoids transformations such as image reconstruction and calculation of Hb states, which potentially can obscure or eliminate information content, especially when nonlinear transformations (e.g., the modified Beer–Lambert law) or filtering steps are involved. By doing this, we are giving up on the opportunity to evaluate the dynamics of Hb states, which may offer insights into physiological processes at play. We opted for this tradeoff because we did not aim to find or explain any specific physiological behavior in tumorous tissue and because our ML approach is agnostic to any underlying physiology. Instead, we prioritized our desire to keep the information content of the data as complete and unobfuscated as possible.

### 6.4 Influence of Channel Density

We trained our classifiers on the full amount of data channels with 32 sources and 64 detectors arranged on each breast. The most successful unilateral and bilateral data representations were also applied to reduced sets of measurement channels that were selected so as to mimic smaller instrumentation setups that would afford reduced source-detector grids of sizes 32×32, 16×16, 8×8, and 4×4 on each breast. Figure 5 plots the classification performance (AUC values) for the different grid sizes. In contrast to the full dataset, for each of the subsets, there exist several distinct spatial source-detector arrangements and, consequently, multiple classification results. There are two results for 32×32 channels, three results for 16×16 channels, and four data points for each of the grid sizes 8×8 and 4×4. We plot these data points individually for unilateral and bilateral classifications and add lines to indicate the respective median values for each grid size.

For unilateral and bilateral classification, the performance shows a tendency to decrease from large toward small grids, as indicated by the respective median lines. In both cases, the spread of the classification results for a given grid size increases from left to right, indicating a greater uncertainty of the achievable results for smaller grids. Presumably, this is because, for smaller grids, the arrangement of the probes relative to the lesion site becomes critical and thus has a stronger impact on the classification result. Because there is no *a priory* information about the tumor location, it is not possible to concentrate a smaller grid on a suspected area.

For all sizes of imaging arrays, we again observe that the unilateral results score systematically lower than the bilateral ones; the green (unilateral) median line stays below its bilateral counterpart for all grid sizes. The data range of the unilateral results (indicated by the green shaded area) is likewise shifted downward with respect to the bilateral results (blue shaded area), although some overlap occurs. Overall, we observe that the minimal classification result for each grid size is always lower for unilateral classifiers and that the maximum classification result for each arrangement is always higher for the bilateral result.

The limited number of data points precludes any deeper statistical analysis so that we base our conclusions on the median results and the extrema.

Inspection of individual data points reveals instances in which the unilateral result exceeds the bilateral classification; however, these are edge cases where the best results of the unilateral classifier trump the worst results of the bilateral classifier. This effect is strongest for the two smallest arrays, where we observe the greatest data spread. However, even for the 32×32 grid, we observe a notable spread of results, with 80% AUC for the lower and 93% AUC for the higher score in the bilateral case. Also, in many instances, the highest scores of the partial grids exceed the result of the full array, in one instance even reaching perfect (100% AUC) classification. This is due to the small datasets we are dealing with and the resulting high variance of the model performance.

The varying results for different probe arrangements of the same size indicate idiosyncratic behavior for each particular probe geometry for the given set of patient data. There is, of course, no *a priori* knowledge of why a certain grid arrangement should be particularly successful (or unsuccessful) for a given measurement, so we cannot expect that a particular way of arranging, say, 16 sources and 16 detectors on the breast will consistently outperform other arrangements of the same size. We rather conclude that sparse arrangements show more inconsistent performance (wider spread) than denser ones and that the only successful strategy to consistently obtain good classification is the use of dense arrays.

In our case, the full grid with 32 sources and 64 detectors per breast yielded results that consistently reached high scores with two different ML methods and were also in agreement with classical statistical methods.

## 7 Conclusions

The results of the present study can only serve as a first indication of the potential of bilateral breast imaging in combination with ML or AI strategies. As a matter of fact, we did not make explicit use of the specifics of the protocol or data collection and did not use any physiological hypotheses or models but instead chose to apply generic methods of representing the raw data in ways that can be fed to our SVM.

We purposely omitted some of the preprocessing steps that are often applied in optical tomography, such as filtering and/or detrending of data. Although these may serve to reduce noise and biases that can have adverse effects on the image reconstruction process, in our case, we decided to leave the data as pristine as possible and not remove any potentially information-carrying signal components. Likewise, we did not exclude any channels from the original data, which often is done in traditional analysis to prune out any noisy channels that may interfere with the analysis or reconstruction.

Although applying such strategies could be explored as a means of excluding noise from the data and potentially further improving classification outcomes, we take the present results as a reasonable estimation of the lower bound of the achievable performance using simple SVM classification. We rather see other routes as more promising to further validate and improve our method, such as employing physiologically informed features or utilizing more sophisticated ML model architectures.

One challenge for ML analysis was dealing with a data set limited to 63 patients, leading to effects such as strongly discretized ROC curves and a lack of reliable sensitivity/specificity measures. Even though a larger patient number would have been desirable, the available data are still a unique and precious trove of bio-optical information well worth examining with modern tools. We fully hope and expect the community to learn from this and to be inspired to further develop and explore the capabilities of bilateral dynamic tomographic breast imaging.

It may be useful for future investigations to evaluate Hb state concentration changes, which can be estimated channel-wise using the modified Beer–Lambert law or in 3D space based on reconstructed local changes of the wavelength-specific absorption. The most straightforward approach would be to calculate feature representations from the basic Hb and HbO concentration changes, which show characteristic behavior in the presence of malignancies.^28^ A more sophisticated, and possibly more promising, approach would be the ML classification based on the hemodynamic state flux metric proposed by Barbour et al.^29^ for the detection of tissue disease states, such as breast cancer.

The ML models used in our study are designed for general-purpose tasks: SVM is a simple linear model, and AutoGluon fits an ensemble of various classical ML models. None of these models exploit the fact that our data are a multi-channel time series within a resting period of the patient. We plan to use this property of the data to design more suitable model architecture in the future, i.e., neural networks with 1D convolutions along the time axis and further layers to combine data across channels.^45^ The results presented in our current paper suggest that even shallow architecture with a few number of parameters could lead to reasonable results. Another avenue for future research is to make use of time-series foundation models^46^ to incorporate prior knowledge of time series.

In summary, we have demonstrated that ML analysis of bilateral dynamical optical mammography can be a very promising candidate for breast cancer detection. Most importantly, we regard the present results as a strong motivation to engage in further studies to further improve and validate our numerical and experimental methods.

## Supplementary Material



## Data Availability

The data presented in this article are publicly available in an Open Science Framework (OSF) repository at the following URL: osf.io/4cr3z/ (DOI https://doi.org/10.17605/OSF.IO/4CR3Z). The code used to generate the results and figures is available in a GitHub repository at the following URL: github.com/ml-lab-htw/optical_mammography_analysis.
