# Supplementary material for: Detection of breast cancer using machine learning on time-series diffuse optical transillumination data
Source: J Biomed Opt. 2024 Nov 11;29(11):115001. doi: 10.1117/1.JBO.29.11.115001 (PMC11552526; doi:10.1117/1.JBO.29.11.115001)
Supplement: Supplementary file 1 [file JBO_029_115001_SD001.pdf]

# – Supplementary material –
Detection of breast cancer using machine learning on time-series diffuse optical transillumination data

Nils Harnischmacher[a], **Erik Rodner**[a], **Christoph H. Schmitz**[b,*]
[a]HTW - University of Applied Sciences Berlin, Faculty II, KI-Werkstatt, Wilhelminenhofstr. 75A, 12459 Berlin, Germany
[b]HTW - University of Applied Sciences Berlin, Faculty I - Health Electronics, Biomedical Electronics and Applied Research (BEAR) Labs, Wilhelminenhofstr. 75A, 12459 Berlin, Germany

Abstract. This supplementary material contains additional details about the breast geometry.

Keywords: optical mammography, diffuse optical tomography, machine learning, breast cancer, mammography.

*Christoph H. Schmitz, christoph.schmitz@htw-berlin.de

1 Breast geometry details

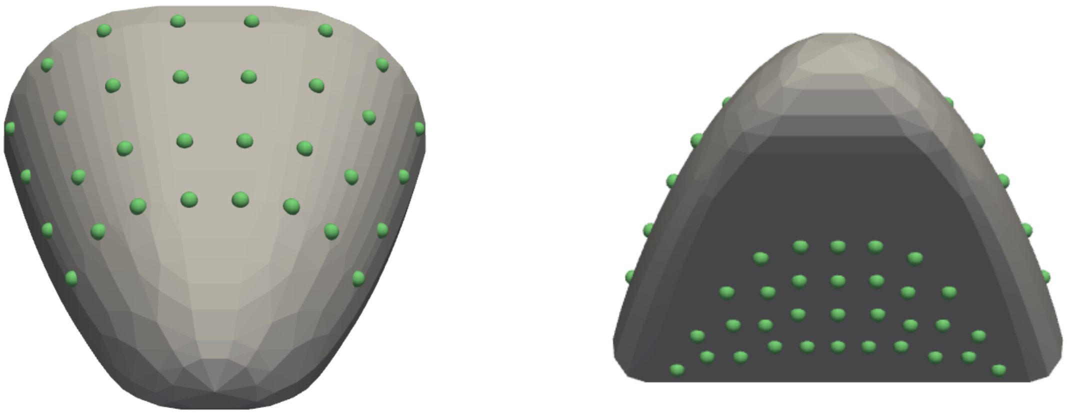

Fig S1: Schematic depiction of the spatial arrangement of the sources and detectors on the breast. Left: View of the upper breast side. Right: View of the breast's underside. The geometry is identical for both breasts (see also Graber *et al.* [28])

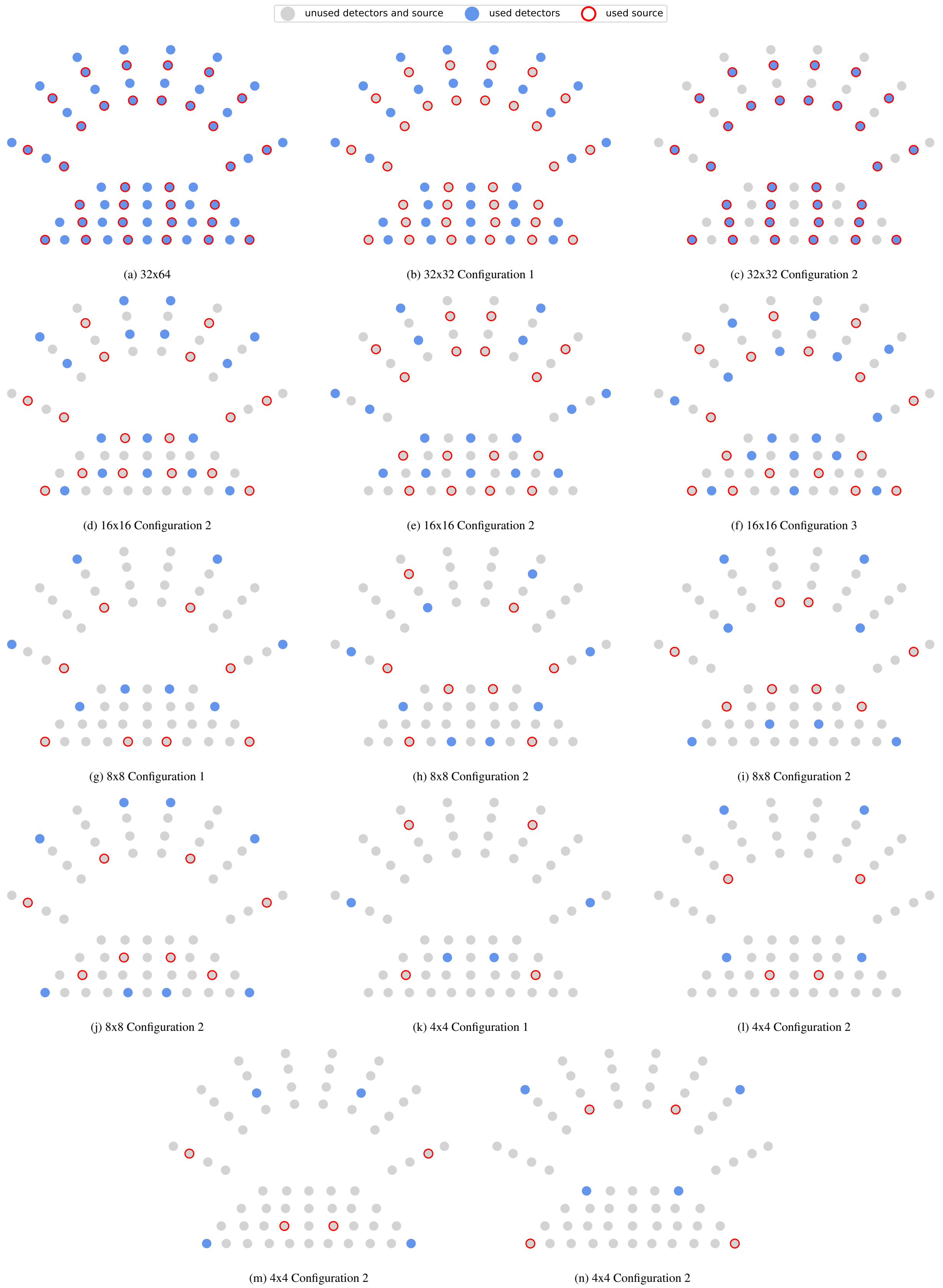

Fig S2: Source/detector configurations used in our sparse channel analysis in a 2D representation of the 3D geometry. The upper half of each plot represents the probe positions on the upper side, and the lower half represents those on the the underside of each breast.
